# Digital Anthropometry for Body Circumference Measurements: European Phenotypic Variations throughout the Decades

**DOI:** 10.3390/jpm12060906

**Published:** 2022-05-30

**Authors:** Marco Alessandro Minetto, Angelo Pietrobelli, Chiara Busso, Jonathan P. Bennett, Andrea Ferraris, John A. Shepherd, Steven B. Heymsfield

**Affiliations:** 1 Division of Physical Medicine and Rehabilitation, Department of Surgical Sciences, University of Turin, 10126 Turin, Italy; chiara.busso@unito.it (C.B.); andrea.ferraris@unito.it (A.F.); 2 Pennington Biomedical Research Centre, Baton Rouge, LA 70808, USA; angelo.pietrobelli@univr.it (A.P.); steven.heymsfield@pbrc.edu (S.B.H.); 3 Paediatric Unit, Department of Surgical Sciences, Dentistry, Gynaecology and Paediatrics, University of Verona, 37126 Verona, Italy; 4 Department of Epidemiology, University of Hawaii Cancer Center, Honolulu, HI 96816, USA; jb99@hawaii.edu (J.P.B.); johnshep@hawaii.edu (J.A.S.)

**Keywords:** anthropometry, avatar, body composition, body shape, waist circumference

## Abstract

This review summarizes body circumference-based anthropometrics that are in common use for research and in some cases clinical application. These include waist and hip circumference-based central body indices to predict cardiometabolic risk: waist circumference, waist-to-hip ratio, waist-to-height ratio, waist-to-thigh ratio, body adiposity index, a body shape index (ABSI), hip index (HI), and body roundness index (BRI). Limb circumference measurements are most often used to assess sarcopenia and include: thigh circumference, calf circumference, and mid-arm circumference. Additionally, this review presents fascinating recent developments in optic-based imaging technologies that have elucidated changes over the last decades in average body size and shape in European populations. The classical apple and pear shape concepts of body shape difference remain useful, but novel and exciting 3-D optical “e-taper” measurements provide a potentially powerful new future vista in anthropometrics.

## 1. Introduction

Anthropometry is the study of the measurements and proportions of the human body. Weight, stature (standing height), length (e.g., limb lengths), skinfold thickness, breadth (e.g., shoulder, wrist, etc.), and circumference are examples of anthropometric measures widely adopted in daily medical practice for monitoring growth and aging as well as for the clinical management of patients.

Circumference measurements can be extremely useful for the management of patients with endocrine and metabolic disorders including people who are overweight and/or obese. For example, waist circumference provides information beyond the body mass index that enables the assessment of (abdominal) obesity-related health risks [1]. Other circumference-based anthropometric indices (described in the next paragraph) can be useful to distinguish between different body-shape phenotypes that exist both among different disorders and between the two sexes. In fact, it is well-known that a visceral fat gradient exists for men (“apple-shaped” because of the larger visceral fat depot) and women (“pear-shaped” because of the larger subcutaneous fat depot). Circumference measurements can also be useful for the management of patients undergoing rehabilitation treatments. For example, the assessment of the upper limb circumference is a common method to estimate the arm volume and quantify the upper limb volume changes in breast cancer survivors with lymphedema [2]. Limb circumference can also provide information about the nutritional status and/or atrophy of one limb with respect to the contralateral limb.

The aim of this review is to provide the main methodological and (patho-)physiological considerations for ensuring the accurate assessment and interpretation of different circumference-based anthropometric indices currently used in research and clinical settings. A better understanding of these techniques, their uses, and their potential is helpful for the prediction, diagnosis, monitoring, and prognosis of multiple disorders and for the proper characterization of pathophysiological mechanisms underlying their sex-specific differences. Another purpose of this review is to present recent developments in imaging technologies and new experimental findings that provide clinicians with tools and resources to easily collect electronic measurements (“e-tape”) with greater rapidity and volume than ever before. Combining the electronic modeling provided by electronic image-scanning with historical anthropometric data enables the generation of visual representations of average European males and females to more clearly highlight how body size and shape have changed over recent decades.

## 2. Assessment of Waist and Hip Circumferences

Waist circumference is a simple measurement that assesses the central (abdominal or android) adiposity. This parameter has emerged as a strong and independent factor for cardiovascular morbidity and for all-cause mortality [1]. Currently, no consensus exists on the optimal protocol for the measurement of this anthropometric variable: the protocols described by WHO [3] and the NIH guidelines [4] are the most commonly adopted in both research and clinical practice. According to the WHO protocol, waist circumference is measured midway between the highest point of the iliac crest and the bottom of the ribcage, while the NIH protocol recommends considering the superior border of the iliac crest to identify the proper measurement location. Absolute differences in waist circumference measurements obtained by the two different protocols are generally small for adult men but much larger for women [1].

Several current definitions of metabolic syndrome include increased waist circumference among the diagnostic criteria but use different cutoff points: the National Cholesterol Education Program Adult Treatment Panel III definition uses the cutoff points of ≥102 cm in men and ≥88 cm in women, while the International Diabetes Foundation definition uses the cutoff points of ≥94 cm in men and ≥80 cm in women [5].

According to the WHO, the waist circumference absolute values can also be used to distinguish between the following three categories of cardiometabolic disease risk: (i) normal risk: ≤80 cm in females and ≤94 cm in males; (ii) increased risk: >80 cm in females and >94 cm in males; (iii) greatly increased risk: >88 cm in females and >102 cm in males [6].

Despite several current definitions of cardiometabolic risk, including waist circumference [5,6,7], this anthropometric parameter is not (yet) widely available in established clinical databases, largely due to the historical use of body mass index in clinical practice [8]. Therefore, Bozeman et al. [9] have previously proposed a model (Table 1, first row) for the waist circumference estimation (from body mass index and demographic covariates such as sex, age, and ethnicity) that can be used to identify individuals at risk for cardiometabolic disease when waist circumference data are unavailable.

The estimated and measured values of the waist circumference are also useful for the anthropometric prediction of lean, fat [10,11], and muscle mass [12,13]. Table 1 also reports recently published equations [10,11,12,13] for the anthropometric prediction (from waist circumference, weight, height, sex, age, and ethnicity) of lean mass, fat mass, and muscle mass: these equations are practical to apply in clinical settings and should be systematically incorporated into the routine management of patients with different health risks or conditions.

Although the waist circumference is a simple measure of abdominal obesity, it assumes that people with the same waist circumference have the same cardiovascular risk regardless of differences in other variables, such as height and fat distribution. However, this assumption is invalid, as body fat distribution is a better predictor of the health hazards of obesity than the absolute amount of body fat and the absolute value of waist circumference. Consistently, individuals with a more android than gynoid body shape tend to have a more adverse metabolic profile and an increased risk for type 2 diabetes and cardiovascular disease. To overcome the limitation of the waist circumference to fully capture cardiometabolic risk, it has previously been proposed to normalize its absolute value to several anthropometric measurements (“appendicular” as well as “central body” anthropometrics). Moreover, given statistical independence information from size and shape (which are both important), the waist and hip circumference-derived anthropometric indices can also be combined to increase the association of basic measures with outcomes of interest.

The waist-to-hip ratio is the dimensionless ratio of the circumference of the waist to that of the hips (i.e., the largest circumference at the buttocks) that is considered a simple measure of peripheral (gluteofemoral or gynoid) adiposity. This anthropometric parameter is considered a valid proxy for central obesity that, according to WHO, should be defined as possessing a waist-to-hip ratio above 0.90 for males and above 0.85 for females [6].

The waist-to-height ratio is calculated as the waist circumference divided by height: this ratio is also independent of units of measurement, provided that the same units are used to measure both the waist circumference and the height. This anthropometric parameter is considered a valid proxy for central adiposity. A boundary value of 0.5 was previously suggested as a risk assessment tool: this translated into the simple message “keep your waist to less than half your height” [14]. The following three risk categories for cardiometabolic disease can be identified (without sex-specific differences) on the basis of the waist-to-height ratio: (i) no increased risk: ratio <0.5; (ii) increased risk: ratio ≥0.5 and <0.6; and (iii) greatly increased risk: ≥0.6 [14]. Previous meta-analyses showed that this anthropometric variable is superior to the body mass index as well as to a matrix using a combination of body mass index and waist circumference for discriminating among different categories of obesity-related cardiometabolic risk [15,16].

Similar to the waist-to-height ratio, an index based on hip circumference and height, namely the “body adiposity index”—BAI = {[hip circumference (cm)/height (m)^½^] − 18}—was also proposed to estimate the body fat percentage for adult men and women of different ethnicities, without numerical correction and without measuring body mass, which can be problematic in certain clinical settings or in locations with limited access to reliable scales [17]. However, Freedman et al. [18] showed that the use of BAI has no advantage over the use of either waist or hip circumference because it can produce biased estimates of body fat percentage (especially in women and in individuals of both sexes presenting body fat percentages greater than 30%).

Thigh circumference (measured at the level of the midpoint on the lateral surface of the thigh, midway between the greater trochanter and the lateral border of the head of the tibia) has long been recognized as a relevant anthropometric measure that identifies individuals with increased risk of premature morbidity and mortality for cardiovascular diseases [19]. This measurement provides an indirect assessment of the amount of subcutaneous fat as well as muscle mass. Hence, the combination of the thigh and waist circumferences could provide information about these two very relevant body sites: for example, an increase in the waist-to-thigh ratio may reflect a relative abundance of central fat, decreased peripheral fat or muscle, or both. The waist-to-thigh ratio has consistently shown a close association with intra-abdominal fat and cardiovascular risk factors [20,21] and was subsequently more associated with type 2 diabetes than other anthropometric parameters [22,23]. Although the waist-to-thigh ratio is an anthropometric index that is simple and practical to apply, its users should recognize that the sex-specific distributions of this ratio may differ depending on the anthropometric protocols used to identify the waist and thigh circumferences, while optimal cutoff points may also vary according to sex and ethnic group. The standardization of methods and sites for measuring waist and thigh circumferences is therefore needed and sex- and ethnic-specific normative values should be identified in future studies.

A body shape index (ABSI) is a measure of the risk associated with waist circumference proposed by Krakauer [24]. It assesses the waist circumference normalized to a standard height and weight and is defined as:

ABSI = waist circumference (m)/[BMI (kg/m^2^)^2/3^ × height (m)^1/2^]

This parameter was able to better predict mortality than waist circumference and body mass index in the Unites States Nutritional Health and Nutrition Examination Survey (NHANES) population [24]. Similarly, the hip index (HI) was proposed by Krakauer [25] as the hip circumference (HC) normalized to a standard height and weight.

It was defined as:

HI (cm) = HC (cm) × (height (cm)/[H])^0.310^ × (weight (kg)/[W])^0.482^

where [H] = 166 cm and [W] = 73 kg, which are average height and weight values. Similar to the body mass index, which compares the body mass among individuals with the same height, ABSI and HI compare the waist and hip circumference, respectively, among individuals with the same body height and weight. Cardiometabolic risk factors and various cancers (including several cancers that are not obesity-related) have been positively associated with ABSI and negatively associated with HI [24,26,27]. Christakoudi et al. [28] have also recently proposed to combine ABSI and HI to define four different body-shape phenotypes: (i) small-ABSI-small-HI individuals (“slim”); (ii) large-ABSI-large-HI individuals (“large”); (iii) large-ABSI-small-HI individuals (“apple”); (iv) small-ABSI-large-HI individuals (“pear”). These different phenotypes were associated with body composition measurements of lean and fat (visceral and abdominal subcutaneous) mass. Moreover, associations between the “apple” phenotype and colon cancer risk were observed in men.

The body roundness index (BRI) was proposed by Thomas et al. [29] as another shape measure that enables the quantification of body shape in a height-independent manner. It can be obtained with the following equation:

BRI = 364.2 − 365.5 × {1 − [(0.5 × waist circumference/π)^2^/(0.5 × height)^2^]}^0.5^

where waist circumference and height are expressed in meters (a simple web-based calculator is available at the following website: https://www.pbrc.edu/research-and-faculty/calculators/body-roundness/, accessed on 28 April 2022). This index serves as a proxy for total and visceral body fat [29]. It shows good discriminatory power for metabolic syndrome in adults of both sexes from diverse populations [30] and is superior to other anthropometric measures for determining the presence of cardiovascular disease risk factors [31]. Moreover, BRI also provides a method to visually compare individual body types and identify the body type location relative to a healthy reference range: briefly, it can be applied as a visual tool for health status evaluations [29].

## 3. Assessment of Limb Circumferences

Limb circumference measurements are frequently used in clinical practice for an indirect evaluation of muscle growth or atrophy (e.g., to assess the effectiveness of strength training or the impact of disuse). For example, the above-mentioned thigh circumference [19], as well as the calf circumference, are considered proxies for muscle mass [32]. Cutoff values for moderately and severely low values of calf circumference were identified as 34 cm and 32 cm in males and 33 cm and 31 cm in females, respectively [33]. Therefore, it has been proposed to include the calf circumference in different sarcopenia screening tools such as the Ishii test [34] and the SARC-CalF questionnaire [35].

Previous studies performed on adult and elderly subjects also show that the calf circumference of the dominant leg can be larger than that of the non-dominant leg [36] and that the asymmetry in lower extremity lean mass is associated with functional mobility [37]. To our knowledge, no normative data exist to distinguish between low (i.e., physiological) and high (i.e., pathological) circumference asymmetry for different limb portions (e.g., thigh, leg). Moreover, calf asymmetry can result not only from muscle disorders but also from soft tissue disorders. In fact, Stein et al. [38] reported that an asymmetry of the calves ≥1 cm may raise suspicion of deep venous thrombosis.

Mid-arm and calf circumferences have also been shown to reflect the nutritional status and predict performance and survival in older adults [39,40,41]. Consistently, the full 18-item version of the widely used Mini Nutritional Assessment includes both circumference measurements that are considered normal for mid-arm values >22 cm and calf values ≥31 cm [42,43].

Limb circumference-based approaches, also known as “circumferential methods”, are extensively used in different clinical settings to obtain estimations of limb volume that are useful for the detection and/or follow-up of peripheral edema [2]. The frustum sign model method and the disc model method represent the most frequently adopted indirect methods. The first approach is based on only two measurements taken at opposite sides of the measured region: the limb volume is approximated by a truncated cone (frustum sign) between the two measurements. The latter approach divides the measured region into several equidistant discs: the limb volume is computed as the sum of the individual disc volumes [2,44,45]. The main advantage of circumferential methods is that they enable a quick estimation of the limb volume and can also be adopted when other methods (such as water displacement or imaging methods) cannot be used [2].

## 4. Digital Anthropometry

Because tape-based body measurements may not be culturally or socially acceptable and also exhibit poor reliability, especially in overweight and obese subjects [46,47,48], there was the need for the development and validation of accessible, valid, reproducible, and cost-effective technologies that are capable of providing robust measurements of lengths and circumferences and body composition estimates [49]. Digital anthropometry by three-dimensional optical imaging systems has recently been shown to provide non-invasive, accurate, and precise measurements of body circumference [49,50]. Multiple three-dimensional optical scanning systems are already marketed to the sports and clothes fitting markets [51,52,53]. Although different implementations of three-dimensional optical technology exist, all commercially available body scanners follow the same three-step process of data acquisition, processing, and anatomical measurements [49]. Data acquisition occurs through either structured light scanning or time-of-flight scanning: briefly, the structured light scanners evaluate light deformation patterns over subjects in view of the cameras, while the time-of-flight scanners measure the round-trip time for reflected photons to travel from the subject in the field of view to the image sensor. Following data acquisition, the raw information is used to create a three-dimensional point cloud that is adopted to obtain an avatar mesh of the human body. Finally, anatomical measurements can be obtained from the avatar using landmarking procedures. In other words, automatic landmark identification allows an “e-tape” measure to be obtained, extracting multiple anatomical measurements [49,50,54,55,56].

**Figure 1:**
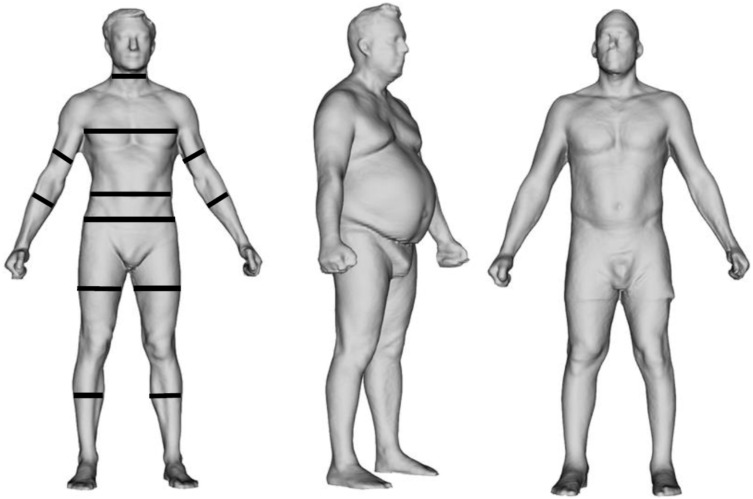
Representative avatars as provided by the software of the Fit3D ProScanner device (hardware version 5.0.6; software version 5.5.0—Fit3D Inc., San Mateo, CA, USA). (**Left panel**) a healthy male subject with standard circumference measurements (continuous black lines) provided by the software. NECK: circumference at mid-point of the neck (over the larynx). CHEST (in males): circumference at inner point of shoulder blades (BUST circumference in females is taken at the forwardmost protruding point above the waist). WAIST: circumference at the small of the back between the lower rib and top of the iliac crest. HIPS: circumference at the rearmost protruding point below the waist. BICEPS: max circumference of the arm between shoulder and elbow. FOREARM: max circumference of the arm between elbow and wrist. THIGH: max circumference of the leg between crotch and knee. CALF: max circumference of the leg between knee and ankle. (**Middle panel**) a male subject with obesity (body mass index: 34.2 kg/m^2^) presenting increased waist circumference (123 cm). (**Right panel**) male patient with congenital inter-limb circumference asymmetries exacerbated following long-term unilateral immobilization of the upper and lower left limbs: right–left biceps: 39.3–33.7 cm; right–left forearm: 31.7–28.3 cm; right–left thigh: 63.1–57.8 cm; right–left calf: 40.9–36.3 cm.

Figure 1 shows representative avatars obtained by a commercially available body scanner that utilizes the structured light scanning technology to investigate a healthy male subject (left panel), a male person with obesity (middle panel), and a rehabilitation patient following the long-term unilateral immobilization of the upper and lower left limbs (right panel). The healthy subject shows standard circumference measurements (continuous black lines) provided by the software of the body scanner. The two patients are presented to show the usefulness of the avatars to quantify the increase in waist circumference (obese patient) and inter-limb circumference asymmetry (rehabilitation patient). We suggest that the systematic incorporation of these “e-tape” measurements into routine patient examinations can be useful for identifying subjects at risk for cardiometabolic and neuromuscular impairment-related comorbidities and for evaluating the effectiveness of pharmacological and rehabilitative interventions.

## 5. European Phenotypic Variations throughout the Decades

A recent study by Wong et al. [57] generated anthropometric measurements in humanoid avatars generated on the basis of body mass, height, age, and waist circumference data from nine NHANES surveys spaced over several decades between 1960 and 1962 and 2015 and 2018. The resulting images show how drastically the average American’s body size and shape have changed over the last 40 years. These findings are consistent with previous studies showing that over the last decades the waist circumference values have increased beyond those expected from the increase in body mass index [58,59].

**Figure 2:**
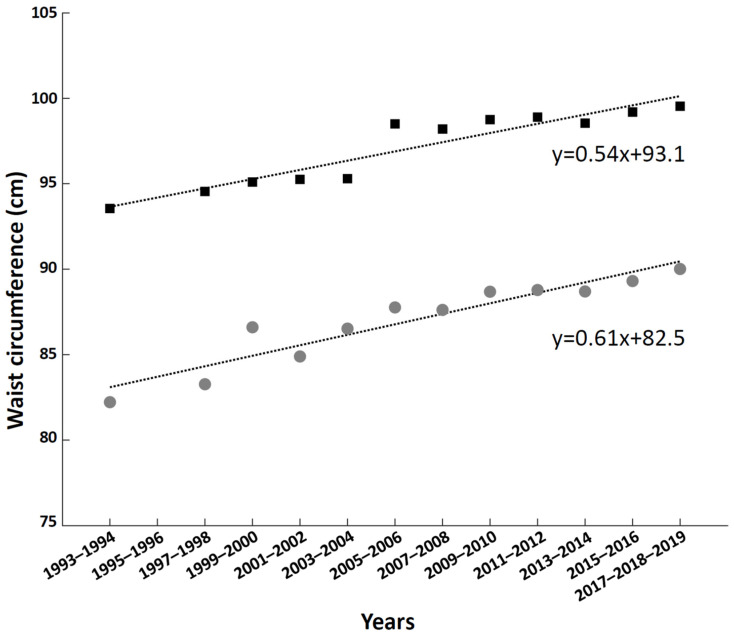
Biannual (triennial only for years 2017–2019) averages of the waist circumference values for young and adult (16–90 years old) men (black squares) and women (grey circles) of the Health Survey for England (HSE) population (between 1993 and 2019—missing data for 1995 and 1996). Data are fit with linear regression lines.

Changes in body size and shape have also occurred within European populations, as indicated by the results of several epidemiological studies performed in different European countries [60,61,62,63,64,65,66]. We report in Figure 2 the time course of the waist circumference obtained during the last three decades from young and adult men and women of the Health Survey for England (HSE) population [67]. The biannual rate of change of the waist circumference was higher in women (0.61 cm/2 years, which corresponds to ~3 cm/decade) compared to men (0.54 cm/2 years, which corresponds to ~2.7 cm/decade).

**Figure 3:**
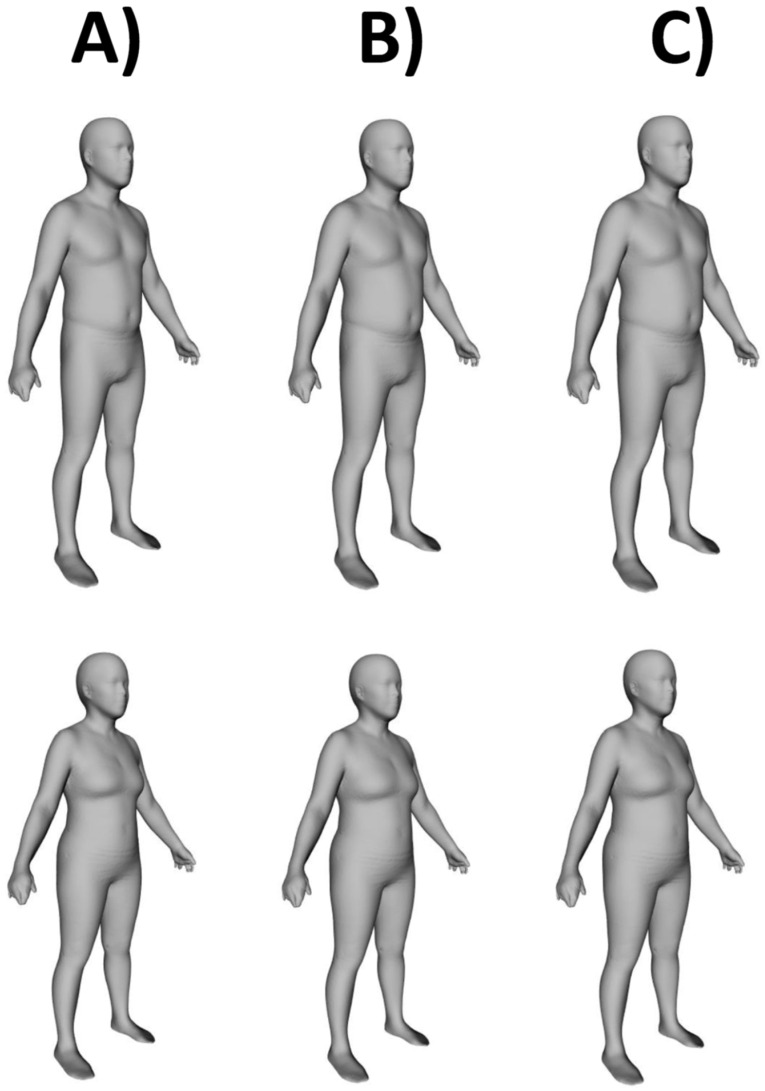
Representative humanoid avatars of average European men (**top row**) and women (**bottom row**) at three time points between 1993 and 2019 as defined by mean population height, weight, and waist circumference. Mean anthropometric data were obtained from the following two cohorts: (i) the Italian cohort of the European Prospective Investigation into Cancer and Nutrition (EPIC-Italy); (ii) Health Survey for England (HSE). Time points: (**A**) Average data from EPIC-Italy 1993–1998 (n = 13,886 men and 31,998 women) and HSE 1993–1994 (n = 12,971 men and 14,677 women) for height, weight, and waist circumference in men (1.73 m, 78.8 kg, 93.5 cm) and women (1.60 m, 65.6 kg, 81.2 cm). (**B**) Average data from EPIC-Italy 2005–2013 (n = 7834 men and 16,892 women) and HSE 2005–2007 (n = 10,041 men and 12,093 women) for height, weight, and waist circumference in men (1.73 m, 81.2 kg, 98.2 cm) and women (1.60 m, 67.8 kg, 86.9 cm). (**C**) Average data from HSE 2017–2019 (n = 6348 men and 8035 women) for height, weight, and waist circumference in men (1.75 m, 86.5 kg, 99.5 cm) and women (1.62 m, 72.8 kg, 90.0 cm).

In order to provide a visual characterization of the European phenotypic variations (i.e., body shape changes induced by body size changes) throughout the decades, we used anthropometric data obtained from the HSE population (period 1: years 1993–1994; period 2: years 2005–2007; period 3: years 2017–2019) [67] and from the Italian cohort of the European Prospective Investigation into Cancer and Nutrition (EPIC-Italy—period 1: years 1993–1998; period 2: 2005–2013) [60,66] to develop the avatar series reported in Figure 3.

Images were generated following the approach outlined by Wong et al. [57]. Briefly, a sex-specific principal component (PC) analysis of the Shape Up! Adults dataset [68,69,70] was performed to derive shape models. Manifold regression analysis utilized the PC shape models to generate a matrix that included the target features for avatar generation. After the conversion of the PC matrix back to the Cartesian space, the weighted sex-specific population mean values of features from the European datasets (HSE and EPIC-Italy) including height, weight, and waist circumference (average values are reported in the caption of Figure 3) were used to generate manifold images for each time period. Manifold regression analysis was performed with the R software (version 4.0.2—R Core Team, 2020: https://www.r-project.org/, accessed on 28 April 2022).

The application of American shape models to European data represents a preliminary analysis that requires the further investigation and expansion of modeling datasets. Still, the body shape changes (i.e., increased “apple-shaped” phenotype in both men and women due to a body weight “shift” from the lower to the upper body, especially to the waist) observed in European adult data resemble those observed in American adults. As properly highlighted by Wong et al. [57], these body shape changes cannot be appreciated when numerical descriptors (such as weight, waist circumference, and body mass index) are considered alone. Therefore, we suggest that this visual analysis of the body shape should be adopted in routine patient management as a counseling tool to increase patient awareness and improve adherence to medication, diet, and physical activity. As recently highlighted by Wells [71], this counseling tool could connect patients and clinicians in new and valuable ways. In fact, people are inherently more interested in how they look to the eye than in the numerical descriptors of their body size and composition.

## 6. Conclusions

Different circumference-based anthropometric indices are currently used in both research and clinical settings for the investigation and management of different disorders. Limb circumference assessments provide an indirect evaluation of muscle size, limb volume, and nutritional status, while several waist and hip circumference-derived indices enable estimations of cardiometabolic disease risk.

The combination of different anthropometric indices and recent technological advances allows for the quantification of body-shape phenotypes that differ between men and women. Moreover, the recent study by Wong et al. [57] and our original findings included above show that body shape phenotypes have changed significantly over the last decades in both American and European people, with this shift reflecting an increased population-wide risk for cardiometabolic disease.

The well-known differences between the body shapes of “apple-shaped” men and “pear-shaped” women remain in use, but a rapid and exciting transformation is taking place that moves anthropometry to the modern age with the introduction of new and interesting biomarkers (“e-tape” measurements) that enable the precise characterization of health status not only at a population level but also at an individual level.

## Figures and Tables

**Table 1:** Equations for the prediction (from anthropometric and demographic data) of waist circumference, lean mass, fat mass, and muscle mass.

Prediction Equations	References
**WC (female)** = 28.81919 + (2.218007 × BMI) + (−3.688953 × AGE CLASS) + (0.125975 × AGE × AGE CLASS) + (−0.6570163 × BLACK) + (0.1818819 × HISPANIC) **WC (male)** = 22.61306 + (2.520738 × BMI) + (0.1583812 × AGE) + (−3.703501 × BLACK) + (−1.736731 × HISPANIC)	[9]
**Lean mass (female)** = −10.683 + (−0.039 × AGE) + (0.186 × HEIGHT) + (0.383 × WEIGHT) + (−0.043 × WC) **Lean mass (male)** = 19.363 + (0.001 × AGE) + (0.064 × HEIGHT) + (0.756 × WEIGHT) + (−0.366 × WC)	[10]
**Fat mass (female)** = 11.817 + (0.041 × AGE) + (−0.199 × HEIGHT) + (0.610 × WEIGHT) + (0.044 × WC) **Fat mass (male)** = −18.592 + (−0.009 × AGE) + (−0.080 × HEIGHT) + (0.226 × WEIGHT) + (0.387 × WC)	[10]
**Relative fat mass (female)** = 64 − [20 × (HEIGHT/WC)] + 12 **Relative fat mass (male)** = 64 − [20 × (HEIGHT/WC)]	[11]
**SM (female)** = 2.89 + (0.255 × WEIGHT) + (−0.175 × HC) + (−0.0384 × AGE) + (0.118 × HEIGHT) **SM (male)** = 39.5 + (0.665 × WEIGHT) + (−0.185 × WC) + (−0.418 × HC) + (−0.0805 × AGE)	[12]
**SM (female)** = (0.25 × WEIGHT) + (0.09 × HEIGHT) + (−0.111 × AGE) + (0.0005 × AGE^2^) + (−0.06 × WC) + (2.0 × RACE) − 4.5 **SM (male)** = (0.47 × WEIGHT) + (0.03 × HEIGHT) + (0.012 × AGE) + (−0.001 × AGE^2^) + (−0.29 × WC) + (1.6 × RACE) + 13.5	[13]

AGE (years); BMI (kg/m^2^): body mass index; HEIGHT (cm); HC (cm): hip circumference; SM (kg): skeletal muscle mass; WC (cm): waist circumference; WEIGHT (kg). HEIGHT and WC are expressed in meters in the equation by Woolcott and Bergman [11] for the relative fat mass estimation. Coefficients for the equation by Bozeman et al. [9]: AGE CLASS (<35 years = 0, ≥35 years = 1); BLACK (no = 0, yes = 1); HISPANIC (no = 0, yes = 1)**.** Coefficients for the equation by Heymsfield et al. [13]: RACE: non-Hispanic white = 0, non-Hispanic black = 1.

## Data Availability

The data that support the findings of this study are available from the corresponding author upon reasonable request.
